# Iron Metabolism, Redox Balance and Neurological Diseases

**DOI:** 10.3390/antiox12091721

**Published:** 2023-09-05

**Authors:** Guofen Gao, Yan-Zhong Chang

**Affiliations:** Ministry of Education Key Laboratory of Molecular and Cellular Biology, The Key Laboratory of Animal Physiology, Biochemistry and Molecular Biology of Hebei Province, Hebei Research Center of the Basic Discipline of Cell Biology, College of Life Sciences, Hebei Normal University, No. 20 Nan’er Huan Eastern Road, Shijiazhuang 050024, China; gaoguofen@hebtu.edu.cn

Iron is essential for life, and the dysregulation of iron homeostasis can lead to severe pathological changes in the neurological system. Iron deficiency slows the development of the neural system and causes mental and emotional disorders [1,2,3], while iron overload is closely related to neurodegenerative diseases such as Alzheimer’s disease (AD), Parkinson’s disease (PD), and cerebral ischemia [4,5,6,7,8]. Free iron can elicit the generation of reactive oxygen species (ROS), due to its ability to catalyze the Fenton reaction [9]. Some free radicals play an important role as signaling molecules in maintaining the normal function of cells, while excessive ROS cause devastating effects on cells, leading to oxidative stress, inflammation, and ferroptosis, contributing significantly to the pathophysiological mechanisms of neurological diseases [10,11,12,13]. In order to protect against harmful effects, the cellular iron content must be precisely controlled, which, at the physiological condition, is tightly regulated by intracellular iron regulatory mechanisms, including hepcidin–ferroportin, transferrin–transferrin receptors, divalent metal transporter 1, ferritin, and iron regulatory proteins (IRP1 and IRP2) [14,15]. Studies have demonstrated that iron dysregulation and redox imbalance are both commonly involved in the occurrence and development of many neurological diseases [11,12,16]. The regulation of the iron metabolism and redox balance has appeared as a potential solution for the treatment of several neurological diseases.

In this Special Issue, “Iron Metabolism, Redox Balance and Neurological Diseases”, five original research articles and five scientific review papers are published. These papers highlight the most recent advances in different aspects of iron regulation and redox imbalance in various diseases, including the molecular mechanisms of iron-induced oxidative damage in disease pathogenesis, potential therapeutic targets and approaches for the regulation of iron metabolism and related damages, and challenges to current studies attempting to understand an aberrant iron metabolism in the pathology of different diseases and its potential clinical applications.

In the original research articles, Han et al. revealed a novel role of iron in the maintenance of cell stemness via the Wnt/GSK-3β/β-catenin signaling pathway [17]. The intermediate molecules that mediated the upregulation of ferritin expression and contributed to stem cell viability and differentiation were also identified. The findings of this study provide a theoretical basis for the development of new strategies based on iron regulation in stem cell treatments for neurological diseases.

Bao et al. designed and characterized a mitochondrial-targeted pseudo-mitochondrial membrane potential (PMMP) constructed by antioxidant MitoQ [18], which selectively protected normal cells from radiation-induced damage in glioma radiotherapy, without affecting the efficacy of radiation in inducing autophagy by regulating the cellular energy supply. This study provides insights into the practical applications of PMMP and antioxidant MitoQ in the selective protection of normal cells and tissues in glioma therapy. This treatment strategy may also be applicable to other neurological diseases.

Liu et al. demonstrated that IRP2 not only regulated cellular iron homeostasis, but also medicated tissue iron distribution by managing the involvement of hypoxia-inducible factor 2 (HIF2) and nuclear receptor coactivator 4 (Ncoa4) [19]. This study highlights that HIF2-NCOA4 is a complex axis that contributes to iron metabolic disorders, including anemia, iron-overload disorder, and neurodegeneration, and provides new target molecules for the treatment of diseases with iron dysregulation.

Han et al. investigated the underlying mechanisms of CY-09, a specific inhibitor of the NOD-like receptor protein 3 (NLRP3) inflammasome, on ameliorating AD classical pathology and cognitive impairment in AD mice [20]. Their findings showed that CY-09 effectively reduced fatty acid synthesis and lipid peroxidation and decreased ROS levels in 3 × Tg-AD mice. However, it had no significant effect on restoring the dysregulation of iron metabolism and ferroptosis. The underlying protective mechanism of CY-09 in AD mice may involve the maintenance of the glucose metabolism and ATP production in the brain. This study provides new evidence for targeting NLRP3 inflammasome as a therapeutic strategy for AD, while the contradiction between decreased ferritinophagy and increased ferroptosis in AD remains to be clarified in future research.

Chen et al. revealed that the dopaminergic neuronal death and Parkinsonian symptoms in OTU domain-containing protein 3 (OTUD3) knockout mice might be caused by activating inositol-requiring enzyme 1α (IRE1α) signaling, which mediated endoplasmic reticulum (ER) stress [21]. The OTUD3 was found to regulate the expression of IRP2. Therefore, knockout of OTUD3 could upregulate the content of iron in dopaminergic neurons in the substantia nigra which, in turn, contributes to increased ER stress, and induces neuronal death and PD pathology. This study provides OTUD3 as a therapeutic target for PD treatment by mediating ER stress to restrain iron-induced apoptosis of dopaminergic neurons.

In the review articles, Luo et al. summarized the molecular mechanisms of ferroptosis in glioma cell growth, invasion, migration, and resistance, and introduced potential applications and challenges of manipulating ferroptosis in the development and treatment of gliomas [22]. They also discussed various nanoparticle-based drug delivery systems, and highlighted the therapeutic opportunities of modulating ferroptosis in glioma treatment to improve clinical outcomes. It was emphasized that, although ferroptosis has great advantages in glioma treatment, further explorations are still needed to assess the advantages and disadvantages of targeting ferroptosis and to evaluate its potential value in clinical applications.

Jiménez-Jiménez et al. systematically reviewed the role of antioxidant coenzyme Q10 (CoQ10) in AD and other dementias [23]. Combined with the use of a meta-analysis, they addressed the concentrations of CoQ10 in different tissues of patients with AD and other dementia syndromes, reviewed the therapeutic response to CoQ10 administration in AD experimental models and patients with AD and other dementias, and discussed the possible therapeutic role of CoQ10. Particularly, the clinical improvement and potential application of mitochondrial activation therapy consisting of CoQ10, iron, and vitamin B6 in AD patients was discussed. Despite the promising neuroprotective effects of CoQ10 detected in different models of AD, further long-term studies with a follow-up period are needed to fill the knowledge gaps regarding both the suitability of CoQ10 as a biomarker of AD and the efficacy of treatments with CoQ10 in patients with AD or other dementias.

Holbein et al. described biological iron requirements, iron regulation, and the nature of iron dysregulation in detail in various disease conditions [24], including viral infections, cancer, ferroptotic cell death, inflammatory diseases, diabetes, cardiovascular diseases, neurological diseases, and so on. They concluded that dysregulated iron homeostasis is a common disease etiology. They also reviewed the current findings pertaining to potential new therapies in these diseases, including iron restriction, iron chelators, hepcidin, and agonists, and proposed the potential application of therapeutics affecting iron dysregulation and lowering excess levels of labile reactive iron in disease therapy. In addition, they identified a number of gaps in the current understanding of iron dysregulation in the pathology of diseases, especially related to cause or effect.

Lee et al. reviewed the interplay between intracellular iron homeostasis and neuroinflammation in neurodegenerative diseases [25]. They introduced the physiological functions of various iron transporters, regulators, and iron-containing enzymes, and summarized the interaction between the aberrant expression and dysfunction of these molecules and inflammation. The interplay shows that maintaining the intracellular iron homeostasis is critical to both the cellular redox balance and steady inflammatory homeostasis. On the contrary, dysregulation of iron metabolism in the central nervous system is commonly associated with neuroinflammation, a crucial hallmark of neurodegenerative diseases. This review article also comprehensively discusses the interactions between iron-related molecules and cell signaling molecules under inflammatory pathological conditions, which may contribute to improving the understanding of neurodegenerative diseases.

Gao et al. summarized and elucidated the interplay between the dysregulation of iron metabolism, redox imbalance, and different neurological diseases [26] such as AD, PD, stroke, abnormal neurodevelopment, and neuropsychiatric disorders. The dysregulation of iron metabolism in each disease, particularly the molecules involved, and the possible mechanisms of iron and oxidative stress in the pathogenesis of these diseases were discussed in detail. This article also reviewed the current progress towards targeting iron metabolism in the treatment of neurological diseases, including the use of iron chelators and iron supplements in different neurological diseases, iron chelators in new administration forms, key molecules in brain iron metabolism as targets, and antioxidants and anti-inflammatory reagents as targets. This review highlights the molecular mechanisms, pathogenesis, and treatment strategies of brain iron metabolism disorders in neurological diseases.

In conclusion, the original research and review articles in this Special Issue provide an updated overview of the advances on the mechanisms or treatments of neurological diseases related to iron dysregulation and redox imbalance. These papers offer fresh perspectives on the expanding knowledge and research possibilities in the field of iron metabolism, redox balance, and neurological diseases, and may stimulate future studies to better target the regulation of brain iron metabolism for the prevention and treatment of neurological diseases.
